# Nanostructured Affinity Membrane to Isolate Extracellular Vesicles from Body Fluids for Diagnostics and Regenerative Medicine

**DOI:** 10.3390/membranes14100206

**Published:** 2024-09-26

**Authors:** Monica Torsello, Margherita Animini, Chiara Gualandi, Francesca Perut, Antonino Pollicino, Cristiana Boi, Maria Letizia Focarete

**Affiliations:** 1 Department of Chemistry “G. Ciamician” and INSTM (National Interuniversity Consortium of Materials Science and Technology) UdR of Bologna, University of Bologna, Via Selmi 2, 40126 Bologna, Italy; monica.torsello@ior.it (M.T.); margherita.animini2@studio.unibo.it (M.A.); c.gualandi@unibo.it (C.G.); marialetizia.focarete@unibo.it (M.L.F.); 2 Interdepartmental Center for Industrial Research on Advanced Applications in Mechanical Engineering and Materials Technology (CIRI-MAM), University of Bologna, Viale Risorgimento 2, 40136 Bologna, Italy; 3 Biomedical Science and Technologies and Nanobiotechnology Laboratory, IRCCS Istituto Ortopedico Rizzoli, 40136 Bologna, Italy; francesca.perut@ior.it; 4 Department of Civil Engineering and Architecture, University of Catania, V.le A.Doria 6, 95125 Catania, Italy; apollicino@unict.it; 5 Department of Civil, Chemical, Environmental and Materials Engineering, University of Bologna, Via Terracini 28, 40131 Bologna, Italy; 6 Interdepartmental Center for Industrial Research on Health Sciences & Technologies (HST) CIRI, University of Bologna, Via Tolara di Sopra 41/E, 40064 Ozzano Emilia, Italy

**Keywords:** electrospinning, regenerated cellulose nonwovens, affinity membranes, extracellular vesicles, regenerative medicine

## Abstract

Electrospun regenerated cellulose (RC) nanofiber membranes were prepared starting from cellulose acetate (CA) with different degrees of substitution. The process was optimized to obtain continuous and uniformly sized CA fibers. After electrospinning, the CA membranes were heat-treated to increase their tensile strength before deacetylation to obtain regenerated cellulose (RC). Affinity membranes were obtained by functionalization, exploiting the hydroxyl groups on the cellulose backbone. 1,4-Butanediol-diglycidyl ether was used to introduce epoxy groups onto the membrane, which was further bioconjugated with the anti-CD63 antibody targeting the tetraspanin CD63 on the extracellular vesicle membrane surface. The highest ligand density was obtained with an anti-CD63 antibody concentration of 6.4 µg/mL when bioconjugation was performed in carbonate buffer. The resulting affinity membrane was tested for the adsorption of extracellular vesicles (EVs) from human platelet lysate, yielding a very promising binding capacity above 10 mg/mL and demonstrating the suitability of this approach.

## 1. Introduction

Cell-to-cell communication is essential for the survival of multicellular organisms. Until the mid-2000s, only two types of communication were known: by direct contact and by secretion of soluble molecules. However, it was discovered that cells can also send signaling packages of molecules wrapped in biological membranes, called extracellular vesicles (EVs), to other cells. These structures are small and transport a variety of biological molecules to target cells, including proteins, RNA, and lipids [1]. EVs are heterogeneous, comprising distinct subpopulations of vesicles that differ in their origin, size, composition, and function. The two main types of EVs are exosomes and microvesicles (MVs). Exosomes are of endosomal origin and are formed by inward budding of the membrane, whereas MVs bud out from the plasma membrane [2]. Although some proteins appear to be enriched in specific EV types, there is significant overlap, and no definitive markers exist to distinguish exosomes from MVs, although exosomes are generally smaller (30–250 nm) compared to MVs (200–1000 nm) [3]. Therefore, the term EVs is used to refer to both according to the indication of the International Society for Extracellular Vesicles (ISEV) [4].

Exosomes, which contain a variety of signaling components such as proteins, lipids, cell surface receptors, enzymes, cytokines, transcription factors, and nucleic acids, are secreted by almost all cell types. These vesicles play a critical role in various cellular functions, such as intercellular communication, cell differentiation and proliferation, angiogenesis, stress response, and immune signaling [5]. Exosomes have been found in nearly all body fluids, regardless of health or disease status. These fluids include urine, blood, serum, breast milk, amniotic fluid, and more.

EVs are receiving tremendous attention from the research community for their potential in cancer treatment and diagnosis, in regenerative medicine, and in drug delivery due to their very low immunogenicity and the stable lipid bilayer that surrounds the vesicles and protects the loaded cargo from the action of native immune cells and digestive enzymes. Furthermore, they are being explored as promising vehicles for targeted drug delivery across the blood-brain barrier (BBB) [6]. Moreover, since exosome secretion occurs under physiological conditions from any type of cell, it is worth mentioning that tumor cells are particularly avid exosome producers. In fact, in patients affected by acute myeloid leukemia (AML), higher levels of exosomal proteins (in the order of mg/mL) are recovered from plasma with respect to healthy donors. Also, AML exosomes have a distinct molecular profile [7], demonstrating the feasibility of using exosomes as biomarkers. In fact, since EV molecular cargo represents the parental cell, EVs can be exploited in the diagnosis of several diseases. A significant portion of the clinical data currently available comes from research on cancer patients, as EVs can serve as valuable tools for monitoring cancer treatment [8,9,10]. Moreover, EVs can be explored in liquid biopsy approaches, which are particularly useful in bone oncology, where tumor sites are frequently not easily accessible and standard biopsies can be very painful [11]. Due to their ability to shuttle intracellularly, exosomes are also considered to have a higher therapeutic potential for various diseases. In fact, exosomes can serve as therapy for their intrinsic cargo or vectors/carrier molecules to elicit a biological response [8,9,12,13].

The isolation of EVs from biological fluids such as blood (plasma or serum) is crucial to allow the analysis of their RNA and protein biomarkers, but this step still represents a difficult task that hinders the full exploitation of their extraordinary properties in biomedicine. The purification of EVs is complicated by the fact that nanovesicles are heterogeneous in size, source, content, and function [14] and are present in biological fluids or matrices composed of different bioparticles. Biological samples contain protein aggregates or lipoproteins that are similar in size and properties to extracellular vesicles [15]. However, in some cases, co-isolated materials are not necessarily considered as “impurities” but as a kind of product enrichment, as they can provide a synergistic effect in combination with EVs [16]. Several methods are currently used by researchers to isolate EVs on a laboratory scale. These methods can be classified according to their mode of operation [17,18,19,20,21]. Traditional methods, based on vesicle size and density, include ultracentrifugation (UC), filtration techniques, size exclusion chromatography, chemical precipitation-based methods (Polyethylene glycol (PEG) precipitation), and microfluidic technologies. UC is considered the gold standard technique for EV isolation, however, it has several drawbacks, such as its time-consuming nature, high cost, the possibility of structural damage to EVs, co-separation of lipoproteins, and its low scalability [19]. Alternatively, techniques based on highly specific interactions with molecules exposed on the surface of EVs, such as affinity chromatography, can be used to isolate exosomes [18,20,21,22,23]. In general, despite the development of various isolation and purification techniques, there are limitations that prevent them from meeting all requirements. In fact, each separation process has advantages and disadvantages and ultimately leads to the isolation of EVs with different characteristics [24,25,26]. For example, in diagnostic applications, it is crucial to utilize methods that provide high EV yields, while maintaining structural integrity can be considered a minor requirement. In contrast, in drug delivery applications, obtaining EVs with intact structures is a priority [27]. A combination of different isolation methods may yield better results than using a single method. Therefore, in order to increase the efficiency and concentration of exosomes, several research groups have started to merge different techniques for exosome isolation and purification, thereby improving yield and purity [18]. Compared to UC, immunoaffinity chromatography (IAC) has been shown to produce comparable results with lower sample volume, providing certain advantages over UC-based methods. This was demonstrated by Zarovni et al. who showed that the yield of exosomes obtained from 400 μL plasma by IAC is identical to that of UC with 2.5 mL samples, showing a significant improvement in isolation efficiency [28]. Affinity separations use antibodies and ligands to separate substances of interest from complex mixtures. In theory, any protein or membrane component present on the membrane of EVs could be used to develop affinity-based EV isolation techniques. Over the past decades, several EV markers have been identified, such as transmembrane proteins, heat shock proteins, fusion proteins (e.g., flotillins, annexins, and GTPases), lipid-related proteins, and phospholipases. Among these, transmembrane proteins, including CD81, CD63, CD9, annexin, and Alix, are the most commonly selected markers for EV isolation. In fact, several commercial EV isolation kits have been developed, such as Exosome-human CD63 isolation/detection (Invitrogen, Waltham, MA, USA) and Exosome Isolation Kit CD81/CD63 (Miltenyi Biotec, Bergisch Gladbach, Germany) [29]. However, classical packed bead columns are not ideal for EV purification due to the large vesicle size (50–200 nm), which does not allow full resin pore accessibility [30].

The use of porous membranes and monoliths could be the ideal solution as they eliminate the need for packing, reduce pressure drop and improve mass transport rates, reduce buffer consumption due to smaller unit dead volumes, and reduce overall processing times which are typical of bead-packed chromatography columns [30].

Affinity membranes are obtained by functionalization of a membrane support with target-specific ligands and represent promising tools for the separation of exosomes. Membrane affinity chromatography combines unprecedented porosity and high surface area with unparalleled selectivity and regenerability [31,32,33]. Indeed, affinity membranes offer rapid processing and high throughput due to their efficient mass transfer capabilities, which stem from the structure of the membrane support. Additionally, the functionalization with EV-specific ligands ensures highly selective binding, reducing the risk of nonspecific adsorption and enhancing the purity of the isolated nanovesicles. Their regenerability allows for multiple cycles of use, significantly lowering operational costs. Furthermore, the structural versatility of nanofibrous membranes, used in our work, enables easy scaling and customization, making them adaptable for various applications in biomedical research and therapeutic exosome production [34,35,36]. Electrospinning is a well-known processing technology that has shown immense potential in creating nanofibrous chromatographic materials with exceptional adsorption capacity, rapid adsorption equilibrium, high solution throughput, and low driving pressure. It is a simple and reliable technique for producing smooth nanofibers with controllable morphology from a wide range of polymers, making it a valuable tool in the protein purification industry [36]. This process involves applying a high electric field to a charged polymer solution or melt and driving the formation of nanofibers through repulsive electrostatic forces. Currently, a variety of electrospun nanofibrous chromatographic membranes have been successfully developed and can be categorized into ion exchange, affinity, and hydrophobic interaction nanofibrous membranes [37]. However, only a few examples of electrospun membranes for the specific purpose of extracellular vesicle purification are available in the literature [38]. Nemeth and colleagues exploited polyvinylpyrrolidone (PVP) electrospun membranes for the preservation of embedded EVs. Akbarinejad et al. developed a system based on electrospun polycaprolactone (PCL) and an electrodeposited terpolymer for the selective capture and release of intact EVs. This platform, functionalized with an aptamer selective for the EV marker CD63, demonstrated the ability to achieve selective and functionality-dependent capture of EV subpopulations [33]. Core-shell PCL-gelatine electrospun nanofibers were developed by Barati and colleagues for the isolation of exosomes and showed an exosome recovery of 87% from the cell culture medium [31].

Cellulose, the most abundant natural polymer on earth, plays a crucial role in tissue engineering, regenerative medicine, drug delivery, and stem cell research due to its renewable, non-toxic, and biodegradable properties. However, its highly crystalline structure poses challenges for direct electrospinning because of its limited solubility in volatile solvents. To address this, cellulose acetate (CA), a cellulose derivative, is often used. CA is readily soluble in various solvents such as acetone, acetic acid, and dimethylacetamide, making it suitable for electrospinning. The resulting nanofibers can be transformed into regenerated cellulose (RC) through a deacetylation process involving an alkali solution and heating. RC combines the beneficial properties of native cellulose, like its hydrophilicity from abundant hydroxyl groups, with enhanced processability. Indeed, RC nanofibers offer desirable mechanical strength, biocompatibility, and a customizable surface chemistry, along with cost-effectiveness and abundance, making them ideal for biomedical applications and scalable production [39,40].

In this study, we present an innovative approach for the adsorption of EVs based on a regenerated cellulose nanostructured affinity membrane prepared by an electrospinning technique and functionalized with an anti-CD63 antibody. The successful membrane preparation and functionalization was demonstrated using Scanning Electron Microscopy (SEM), Differential Scanning Calorimetry (DSC), X-ray Photoelectron Spectroscopy (XPS), and fluorescence microscopy. Finally, a proof of concept of the suitability of the affinity membranes for EV capture was obtained by adsorption of extracellular vesicles from platelet lysate, a purified solution that demonstrates the suitability of this approach due to the specific affinity of the anti-CD63 antibody for the tetraspanin protein CD63 present on the EVs surface.

## 2. Materials and Methods

### 2.1. Materials

Cellulose acetate (CA), (MW 74,000 g/mol, DS 3.0), was purchased from Fluka Milan, Italy. CA ds 2.4, CA ds 1.8, and CA ds 0.8 were purchased from Eastman Chemical Company, Langenfeld, Germany. Dichloromethane (DCM), Ethanol (EtOH), Acetone (Act), Hydrochloric acid (HCl), Sodium Hydroxide (NaOH), 1,4-Butanediol-diglycidyl ether (BDDE), Sodium Bromide (NaBr), Sodium Hypochloride solution (NaClO), Sodium Chlorite (NaClO_2_), 1-Ethyl-3-(3-dimethylaminopropyl)carbodiimide (EDC), N-Hydroxysuccinimide (NHS), 1,4-Diaminobutane (DAB), 2-(N-morpholino)ethanesulfonic acid, Sodium Chloride (NaCl), Potassium Chloride (KCl), Disodium phosphate (Na_2_HPO_4_), Monopotassium phosphate (KH_2_PO_4_), Sodium carbonate (Na_2_CO_3_), Fluorescein isothiocyanate (FITC) were purchased from Sigma Aldrich, Milan, Italy. Acetic acid glacial (AcOH) was purchased from Carlo Erba Reagents, Milan, Italy and sodium borohydride (NaBH_4_) was purchased from Merck, Milan, Italy. The LAMP-3/CD63 Antibody, FITC conjugate, and Bicinchoninic Acid Protein Assay (BCA) were purchased from Thermo Fisher, Segrate (MI), Italy.

### 2.2. Membrane Preparation and Functionalization

#### 2.2.1. Preparation of Regenerated Cellulose Electrospun Nanofiber Membranes

The electrospinning setup, placed in a glove box (Iteco Eng. Ravenna, Italy, 100 × 75 × 100 cm), was composed of an SL 50 P 10/CE/230 high voltage power supplier (Spellman, New York, NY, USA), a KDS syringe pump (KDScientific Inc., Holliston, MA, USA), a glass syringe (diameter 14.55 mm) for the polymeric solution, a stainless-steel blunt-ended N-P3-G18 needle (Hamilton, Bonaduz, Switzerland) connected with the power supply electrode, and a grounded aluminum plate to collect the electrospun fiber mesh. The glass syringe was filled with the polymeric solution. Several CA solutions were prepared to optimize the fiber production, varying polymer concentration and DCM/EtOH ratio, as discussed in the Results and Discussion section and by dissolving the proper amount of polymer at room temperature (RT) in a determinate solvent ratio. Electrospun membranes were eventually produced from a 6% *w*/*v* CA solution in DCM/EtOH (80/20 *v*/*v*). The electrospinning process was performed at a flow rate of 2.0 mL/h and the applied voltage was 15 kV. The aluminum collector was 15 cm away from the needle of the syringe. The needle’s inner diameter was 0.51 mm. Fiber production was carried out at RT and relative humidity in the range of 30–70%. The heat treatment of the electrospun CA nanofibers was performed by sandwiching the mat with two plane poly(tetrafluoroethylene) plates and putting it into a laboratory press (Carver) at 180 °C for 1 h. The deacetylation of CA allows the regeneration of the cellulose structure. The process was performed by dipping CA into a 0.1 M NaOH solution in H_2_O/EtOH (4:1) mixture for 24 h at RT, as previously reported [41]. The fibers were rinsed twice in deionized (DI) water for 20 min and then dried on a filter paper at RT for 30 min before the final drying in the desiccator under vacuum for 24 h.

#### 2.2.2. Epoxidation of Regenerated Cellulose Nanofiber Membranes

The epoxidation reaction introduces epoxy moieties on the surface of the fibers. A sample of RC (0.59 mmol), was placed in a round bottom flask under gentle shaking. Then 10 mL of NaOH aqueous solution 0.6 M, 60 mg (2 mg/mL) of NaBH_4_, and 10 mL (52 mmol) of BDDE were added respecting this order. The suspension was stirred at RT for 6 h. The reaction was quenched with two washings with DI water and four washings with 50 mL of Act. The sample was finally placed in the desiccator under vacuum.

#### 2.2.3. Ligand Immobilization

The bioconjugation of the fibers was performed in a multi-well plate filled with circular samples of RC cellulose functionalized with BDDE. Two different anti-CD63 concentrations were investigated, namely 1.6 μg/mL and 6.4 μg/mL. Negative controls were obtained by using untreated RC or by omitting the anti-CD63 on the functionalized mat in order to evaluate the non-specific binding of the biomolecule to the fiber surface. The membranes were preliminary rinsed for 5 min in MES buffer. Then, they were incubated with the two different concentrations of anti-CD63 and were left gently stirring overnight at 4 °C. Finally, they were re-suspended in phosphate-buffered saline (PBS).

### 2.3. EV Isolation from Human Platelet Lysate (PL)

Human platelet lysate (PL) is generated from buffy coats by means of a freeze-thaw procedure. According to Fernandez-Rebollo et al., platelet units, collected after their expiration date (5 days after harvesting), are aliquoted (40 mL), twice frozen at −80 °C, re-thawed at 37 °C, and centrifuged 2600× *g* for 30 minutes at RT to remove cell fragments [42]. The supernatant was then transferred into new tubes, and 2 U/mL heparin (Epsoclar, Mayne Pharma, Naples, Italy) was added before EV isolation to prevent coagulation. The study was conducted on fully anonymized expired platelet unit and was authorized by the Centro Regionale Sangue dell’Emilia Romagna (Prot 31780/10-14).

EVs were then isolated by serial low-speed centrifugation followed by ultracentrifugation, as previously described [43]. Briefly, PL was centrifuged: 500× *g* for 10 min (two times), 2000× *g* for 15 min (two times), and 10,000× *g* for 30 min (two times) (F34-6-38 rotor, Eppendorf 5810R, Eppendorf, Milan, Italy) at 4 °C to remove cellular debris. The supernatant was then ultracentrifuged at 30,000 rpm for 1 h at 4 °C (Ti 45 rotor, Beckman Coulter, Rome, Italy). The EV pellet was washed once in PBS and centrifuged at 30,000 rpm for 1 h at 4 °C (Ti 70 rotor, Beckman Coulter, Milan, Italy). The EV pellet was re-suspended in sterile PBS and stored at −80 °C until use.

### 2.4. EVs Adsorption onto Affinity Membranes

The adsorption of the EVs was performed on a batch system by incubating the membrane samples in a 24-well plate: one membrane disc of 1.0 cm of diameter was inserted in a well containing 300 μL of EV solution at a concentration of 0.34 μg/mL for 3 h at 4 °C. The membrane tested included the affinity membranes made by epoxidized RC bio-conjugated with 1.6 μg/mL and with 6.4 μg/mL of anti-CD63, the native, non-epoxidized RC membranes with anti-CD63, and the epoxidized RC non bio-conjugated. Additional negative controls consisted of incubating all membrane types in PBS buffer instead of EVs. The amount of EVs adsorbed on the membranes was measured with the Bicinchoninic Acid (BCA) protein assay. A calibration curve was made by diluting the bovine serum albumin (BSA) standards from the 1 mL ampules provided with the commercial kit. After 3 h of incubation, every membrane sample was removed from the 24-well plate and the total protein concentration was measured for all samples. The analysis was first performed on the feed EV solution to quantify the initial concentration and both on the final liquid solution after adsorption and directly on the membranes as described by Boi et al. [44]. After incubation at 37 °C for 30 min with the BCA working reagent, the absorbance signal of each sample was measured spectrophotometrically by UV readings at 562 nm (Shimadzu, Kyoto, Japan).

### 2.5. Characterization Techniques

Thermogravimetry (TGA) (TGA2950 thermogravimetric analyzer, TA Instruments, New Castle, DE, USA) and differential scanning calorimetry (DSC) (Q100 DSC apparatus, TA Instruments, New Castle, DE, USA) measurements were performed in order to assess the success of the regeneration process and to establish the heat treatment temperature of the CA fibers. The morphology of the fibers was characterized by scanning electron microscopy (SEM) (Philips 515), after gold sputtering. The distribution of fiber diameters was determined by measuring about 100 fibers and the results are given as the average diameter ± standard deviation of the mean (SD). The Student’s *t*-test was used to verify the statistical significance of the difference between the mean values (*p* < 0.05). Contact angle measurements of CA and RC membrane were performed using an optical contact angle meter (KSV Instruments CAM 101, bought from Alfatest, Cernusco sul Naviglio (MI), Italy) with CAM software, by recording the side profiles of deionized water drops for image analysis. Five drops were observed on different areas for each sample, and contact angles were reported as the average value ± standard deviation. The ATR-FTIR spectra of the CA and RC membrane were obtained on a Bruker Alpha infrared spectrometer equipped with a transmission sample compartment. The samples were placed in NaCl cells and the data were analyzed with OPUS software. In order to establish the resulting chemical changes of the material after the regeneration process, the Surface chemical composition of as-synthesized and modified mats was determined by XPS using a VG Instrument electron spectrometer equipped with a Mg K_α1,2_ X-ray source (1253.6 eV) and a CLAM II analyzer. The X-ray source under standard conditions was at 200 W, 10 kV, and 20 mA. The base pressure of the instrument was 5 × 10^−10^ Torr and an operating pressure of 2 × 10^−8^ Torr was adopted. Widescan acquisitions were carried out using a pass energy of 100 eV, while narrow scans were acquired using a pass energy of 50 eV. Semi-quantitative surface analyses were performed by determining photoelectron peak areas obtained by multiplying the experimental values with the appropriate sensitivity factor. A spectral acquisition take-off angle (t.o.a) of 45° was used. Considering that the relationship between the sampling depth (*d*) and the t.o.a (θ) is represented by the equation d = 3λ sin θ, where λ is the inelastic mean free path of the photoelectrons (for carbon λ = 14 Å) [45], the thickness of analyzed layers is about 30 Å. The calculation of the areas corresponding to the different photoelectron peaks was performed using VGX900x software; the curve fitting elaborations were determined by PeakFit software (version 4, from SPSS Inc., Chicago, IL, USA). The curve fitting of the C_1s_ envelope was determined as the product of Gaussian and Lorentzian functions (80:20): the full width at half maximum of each curve was kept equal to 1.9 ± 0.1 eV. Binding energies referred to the C-H level at 285 eV. The amount of anti-CD63 immobilized on the surface of the epoxidized membranes was characterized by fluorescence imaging performed with a Nikon fluorescence microscope (Chiyoda, Tokyo, Japan). The images were collected with a mercury lamp with a FITC filter (Exc = 465–495 nm Em > 505 nm) by setting the following operative parameters: dichroic mirror = 505 nm, exposition time = 500 ms, and Gain = 3. The software used for the acquisition was HC-Image (Hamamatsu Corporation, Bridgewater, NJ, USA). The mean values of the relative fluorescence intensity data were calculated with the software ImageJ 1.53 k (National Institutes of Health, Stapleton, NY, USA) on a 50 × 50 pixels square area.

## 3. Results and Discussion

### 3.1. Preparation and Characterization of Electrospun Cellulose Nanofibers

#### 3.1.1. Electrospinning of Cellulose Acetate Solutions

CA was used to prepare electrospun nanofiber mats, due to the limited solubility of cellulose which is an obstacle to its processing through solution electrospinning. Four CA polymers with different degrees of substitution (ds) were tested, namely CA ds 3.0 (fully substituted), CA ds 2.4, CA ds 1.8, and CA ds 0.8, and their solubility in DCM/EtOH was evaluated. From solubility evaluation tests it was demonstrated that only CA ds 2.4 and CA ds 3.0 were soluble in the DCM/EtOH 90:10 binary solvent system. Therefore, they were chosen to prepare the solutions for the electrospinning process.

Optimization of the electrospinning process aims to achieve continuous bead-free fibers through the appropriate selection of the polymer solution and processing parameters. None of the variables influence the process independently, and this interplay of roles leads to a trial-and-error experimental approach. Fiber mats were prepared from solutions of CA ds 3.0 and CA ds 2.4 at different concentrations and solvent ratios. The objective was to observe how these parameters could influence the size and morphology of the fibers. DCM/EtOH ratios of 90/10, 80/20, and 70/30 (*v*/*v*) and CA concentrations of 5%, 6%, 8%, and 10% (*w*/*v*) were used on the basis of a previously reported study [45,46]. Table 1 summarizes the electrospinning experiments performed for process optimization and the results obtained. From the first electrospinning trials, it was evident that CA ds 3.0 gave better results in terms of process stability and nanofiber morphology than CA ds 2.4. Therefore, the electrospinning process was optimized for CA ds 3.0 which enabled the production of fibers with no beads.

**Figure 1:**
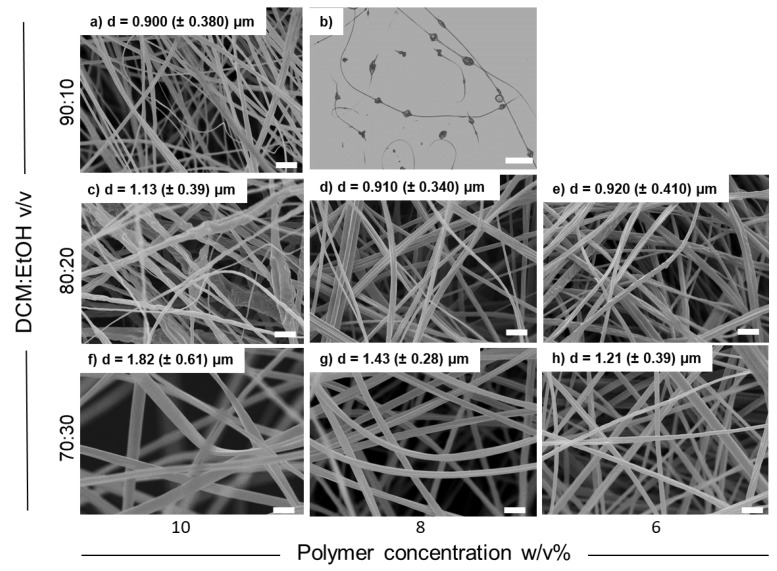
SEM images of CA (ds = 3.0) samples electrospun from different starting solutions: (**a**) 10 *w*/*v*% in DCM:EtOH = 90:10 (*v*/*v*), (**b**) 8 *w*/*v*% in DCM:EtOH = 90:10 (*v*/*v*) (optical microscope image), (**c**) 10 *w*/*v*% in DCM:EtOH = 80:20 (*v*/*v*), (**d**) 8 *w*/*v*% in DCM:EtOH = 80:20 (*v*/*v*), (**e**) 6 *w*/*v*% in DCM:EtOH = 80:20 (*v*/*v*), (**f**) 10 *w*/*v*% in DCM:EtOH = 70:30 (*v*/*v*), (**g**) 8 *w*/*v*% in DCM:EtOH = 70:30 (*v*/*v*), and (**h**) 6 *w*/*v*% in DCM:EtOH = 70:30 (*v*/*v*). SEM scale bar = 5 µm; optical microscope scale bar = 20 µm.

Morphological analysis of the fiber mats produced with CA ds 3.0 showed that the variation of the electrospinning parameters (such as flow rate, voltage, and needle-to-collector distance) did not correspond to significant changes in fiber diameter. On the contrary, the decreased viscosity of the solution at lower polymer concentrations translated into the electrospinning of continuous fibers with a smaller diameter (compare Figure 1f with Figure 1g,h as an example). However, when lowering the CA concentration, a decrease in solvent ratio was necessary to avoid the formation of beads. Indeed, a progressive disappearance of beads and an increase in fiber diameter was observed in going from 90/10 *v*/*v* to 70/30 polymer/solvent ratio (compare Figure 1b with Figure 1d,g). The best combination obtained during the process optimization of CA ds 3.0, both in terms of fiber diameter and jet stability, was recognized to be the one prepared with a solution at 6% *w*/*v* in DCM/EtOH 80/20, which was chosen as the most suitable material to perform the next steps of the work, starting from CA deacetylation to obtain RC fibers.

#### 3.1.2. Morphological Characterization of the Membranes

**Figure 2:**
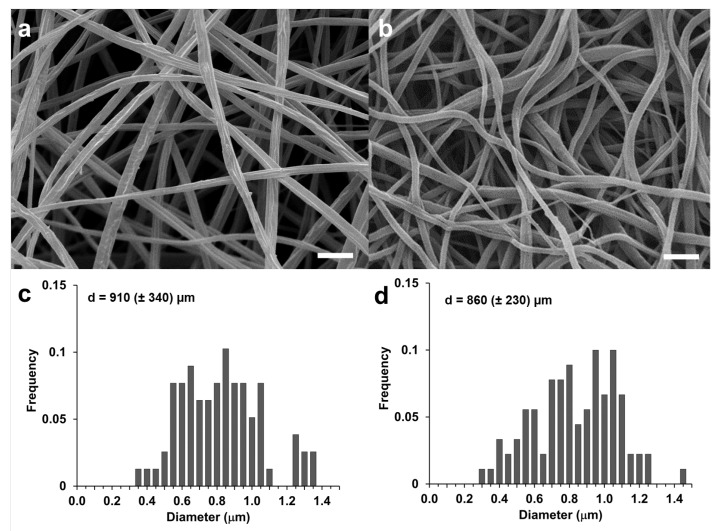
SEM images of CA (**a**) and RC (**b**) fibrous mats (scale bar = 5 μm); fiber diameter distribution of CA (**c**) and RC (**d**).

Bead-free nanofibers can be observed in the SEM images shown in Figure 2, where a comparison between a CA with an RC membrane can be easily done. The absence of fused junctions at the contact point between fibers explains the fluffy cotton appearance of the electrospun mat. The uniformity of the diameters of the CA fibers observed in Figure 2a was confirmed by the dimensional analysis of the fiber diameters that gave a mean diameter of 910 (±340) µm (Figure 2c). In line with previously reported results [41], the regeneration process did not change the morphology of the electrospun material and the fiber diameter was not significantly different (Figure 2d, *p* > 0.05).

#### 3.1.3. Verification of the Effectiveness of Cellulose Regeneration

**Figure 3:**
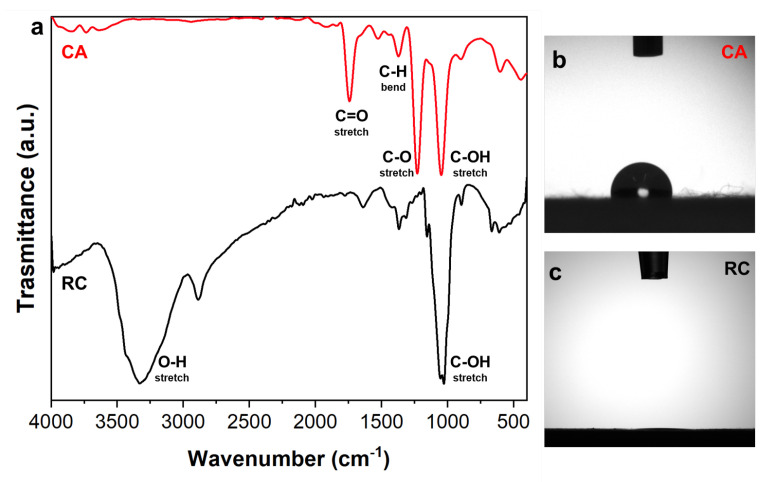
(**a**) Comparison of the ATR-FTIR spectra of cellulose acetate (CA, red) and regenerated cellulose (RC, black); water drop profiles on CA (**b**) and RC (**c**) electrospun samples.

The success of the cellulose regeneration process was assessed by ATR-FTIR spectroscopy and results are reported in Figure 3a, where the spectra of untreated CA and RC electrospun mats are compared. The characteristic peaks of CA appear at 1050 cm^−1^ (C-OH stretching), 1240 cm^−1^ (ester C-O stretching), 1380 cm^−1^ (C-H bending), and 1760 cm^−1^ (C=O stretching). The success of the regeneration process is confirmed by the disappearance of the acetyl group peak at 1240 cm^−1^ and 1760 cm^−1^ and the appearance of a broad absorption peak at 3330 cm^−1^, due to the hydroxyl group stretching in the spectrum of the RC mat as shown in Figure 3a, as previously reported [41].

Similar conclusions can be drawn by water contact angle measurements that were used to confirm the conversion of ester groups into hydroxyl groups leading to a greater water wettability. The hydrophobicity of the untreated CA electrospun mat is confirmed by the average angle of 106 ± 3° (Figure 3b). On the other hand, the water drop was suddenly adsorbed by the membrane when falling on the surface of RC fibers (Figure 3c). The increased wettability of the CA mats when deacetylated, once again provided evidence of the success of the regeneration process.

**Figure 4:**
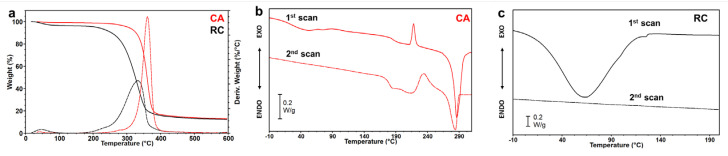
Thermal characterization of CA and RC electrospun mats: (**a**) comparison of the TGA (solid lines) and DTGA (dashed lines) measurements for CA (red) and RC (black) mats; (**b**) and (**c**) DSC measurements (first heating scan and second heating scan after quenching) of CA and RC electrospun mat, respectively.

The thermal stability of CA and RC electrospun mats was evaluated through TGA measurements. Figure 4a shows that the CA membrane was stable up to around 300 °C, after which it showed a single weight loss centered at a temperature of maximum degradation rate (T_max_) around 360 °C. A residue of about 13% of the initial weight was detected at 600 °C. TGA results of RC (Figure 4a) showed that for this polymer, the main thermal degradation step started around 200 °C, earlier than CA, with a single weight loss centered at a T_max_ around 334 °C. This result aligns with previous studies that reported higher thermal stability for cellulose acetate compared to native cellulose [47,48], which has a chemical structure similar to that of regenerated cellulose. A 13% residue of the initial weight was detected at 600 °C. Moreover, both samples showed a weight loss between RT and 100 °C, whose entity was 0.65% for CA and 6.2% for RC, which was attributed to adsorbed water evaporation. The different amount of water loss of the two samples was attributed to the higher hydrophilicity of RC with respect to CA, confirming the WCA results.

The calorimetric characterization of the materials is shown in Figure 3b,c. The first DSC scan of electrospun CA (Figure 4b) shows an endothermic broad peak centered around 50 °C, attributed to the loss of water, followed by a step change of the baseline ascribable to the glass transition temperature at 187 °C, an exothermic peak at 218 °C, attributed to a crystallization process, and an endothermic peak at 286 °C, corresponding to the melting of the crystalline phase. The difference between the areas under the melting and the crystallization peaks (∆H = 18.20 J/g) suggests that during the electrospinning process, a crystalline phase was developed. The second scan, collected after the quench, showed a glass transition at 178 °C and a crystallization and melting peak, centered respectively at 235 °C and 284 °C, of the same entity, thus suggesting that the quenching process was efficient and an amorphous polymer was obtained after the rapid cooling. The DSC scan of the RC electrospun mat (Figure 4c) showed a highly crystalline polymer, which, according to the literature [31] degraded before melting. Therefore, the DSC curves exhibited neither a T_g_ nor a T_m_. The broad endothermic peak centered at 60 °C and corresponding to the loss of water was found to be more intense for RC than CA electrospun mat, given the higher hydrophilicity and the higher specific surface area of RC fibers than CA fibers, which contributed to higher adsorption of water.

**Figure 5:**
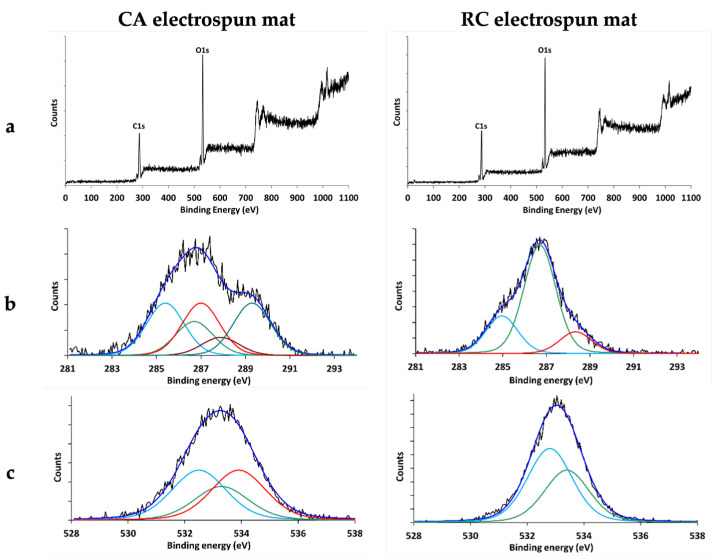
XPS spectra of CA and RC electrospun mats: (**a**) widescan (**b**) C1s envelope and its components (**c**) O1s envelope and its components.

XPS measurements were carried out to assess the success of the cellulose regeneration process. The widescan of the CA and RC electrospun mat (Figure 5a) revealed the presence of Carbon (peak centered at 285 eV) and Oxygen (peak centered at 532 eV). The experimental atomic ratio O/C for CA mats was equal to 0.69, corresponding to the following atomic composition: carbon = 59.1% and oxygen = 40.9%. The theoretical atomic percent abundance obtained from the empirical formula C_12_O_8_ of the CA repeating unit, calculated considering that hydrogen is not detected by XPS, is C = 60% and O = 40%. It can therefore be concluded that the experimentally obtained atomic percent abundance values are in good agreement with the calculated ones. The curve fitting analysis performed on the C1s and O1s peaks is also in agreement with the structure of CA. Considering the structure of the CA repeating unit, the presence of five chemical environments for carbon can be predicted, listed in order of increasing binding energy: 3 carbon atoms in α positions to carboxylic functions, 2 carbon atoms involved in single bonds with oxygen, 3 carbon atoms bonded to oxygen atoms in ester groups, 1 carbon atoms bonded to two oxygen atoms, and 3 carbon atoms involved in carboxylic functions. As shown in Figure 5b, the curve fitting analysis was performed using peaks whose positions (285.4, 286.6, 287, 287.9, and 289.3 eV) and area ratios (25:16:25:9:25, respectively) are in good agreement with the expected values. The curve fitting analysis conducted on the O1s peak also revealed the three expected chemical environments (peaks) with respective area ratios of 37:25:37 (Figure 5c), confirming the structure of the mats’ surface. The experimental atomic ratio obtained for RC electrospun mat O/C = 0.66, corresponding to the following atomic composition: carbon = 60.3%, Oxygen = 39.7%. Taking into account that hydrogen is not revealed by XPS, the formula of the RC is C_6_O_5_, which means that both the atomic ratio and the atomic percentage composition were quite far from the expected theoretical values, respectively, 0.83 and C = 54.5% O = 45.5%. This could be explained by observing the curve fitting elaborated on the C1s peak (Figure 5b): in addition to the signals expected for the structure of the repeating unit of the cellulose (the peak centered at 286.7 eV corresponding to the 5 C atoms involved in the single bond with the oxygen, and the peak centered at 288.2 eV corresponding to the C atom bonded to the two atoms of oxygen in the glucose ring) there was a signal centered at 285.0 eV due to hydrocarbon contamination usually present in samples analyzed by XPS. This was responsible for the low atomic ratio value of O/C. By eliminating the contribution of this hydrocarbon contamination, evaluated by curve fitting at approximately 22% of the total area, the O/C ratio resulted in 0.85, and the atomic percentage composition C = 54.1% and O = 45.9%. Moreover, the ratio between the areas of the two peaks centered at 286.7 eV and 288.2 eV, respectively, was equal to five in perfect accordance with the structure of the repeating unit of RC. The curve fitting of the O1s peak confirmed the presence of the two chemical states of the O atom: one centered at 532.8 eV corresponding to the three O atoms of the hydroxyl group and one centered at 533.4 eV corresponding to the two O atoms, one of which is in the ring of the glucose and the other one forms the glycosidic bond between two units of glucose. These results fit the expected values for the total conversion of the CA to RC well and demonstrate that the deacetylation treatment applied in this work was successful in producing RC fibers, in line with previous results [41].

### 3.2. Functionalization and Bioconjugation of Regenerated Cellulose Nanofiber Membranes

Chemical activation and ligand coupling are some of the possible strategies available for the preparation of affinity membranes. It is well known that carboxyl (-COOH), primary amine (-NH_2_), sulfhydryl (-SH), and carbonyl (RCOR′) groups are the moieties most commonly involved in the coupling with the affinity ligands [49]. Despite their widespread presence in several polymers, a prior activation step is sometimes required to introduce them to the surface of the material. Moreover, functionalization allows us to overcome the problem of low steric accessibility of the ligands when directly bound to the surface since it introduces sites for further interactions with spacer arms and ligands. It allows the oriented and ordered coupling of biomolecules, facilitating the performance of their biological functions.

#### 3.2.1. Membrane Activation with Epoxy Groups

**Figure 6:**
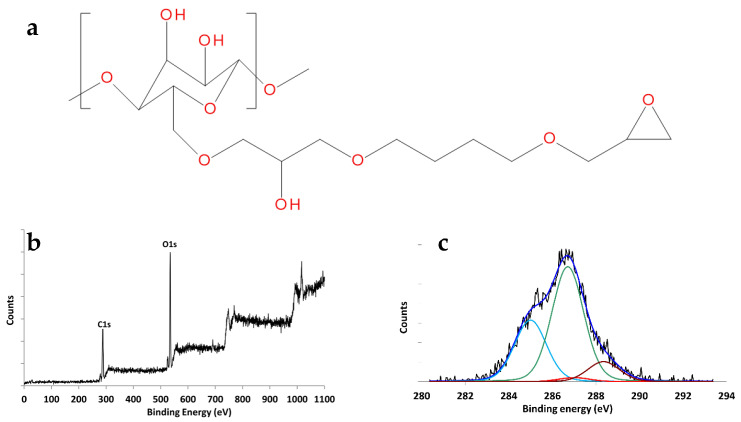
(**a**) Chemical structure of RC functionalized with BDDE; (**b**) XPS widescan and (**c**) C1s envelope of functionalized RC.

Epoxy groups were introduced on the membrane surface by reacting the RC mats with BDDE under reducing conditions (NaBH_4_) as shown in the reaction scheme of Figure 6a [50]. BDDE has the double function of introducing the highly reactive epoxy groups and of creating a spacer arm, between the membrane fibers and the ligand molecules to favor ligand mobility and accessibility under binding conditions. The choice of epoxy chemistry was due to previous experience in our laboratories [51]. Indeed, we tested also the TEMPO immobilization chemistry, which was less efficient and was abandoned after a few initial tests. The success of the membrane modification was assessed by XPS analysis as shown in Figure 6b,c.

The XPS widescan of the RC treated with BDDE (Figure 6b) showed the presence of Carbon and Oxygen. The detected O/C atomic ratio was 0.60. If the treatment led to a complete conversion of the primary alcohol groups, there should be four different chemical environments for carbon: (i) two hydrocarbon carbon atoms with a signal expected to fall at 285.0 eV, (ii) eleven carbon atoms involved in single bonds with oxygen, whose signal should therefore fall at ~286.7 eV, (iii) two carbon atoms involved in the epoxide function, whose signal should fall at ~287.0 eV, (iv) one carbon atom involved in two bonds with oxygen, whose signal should fall at ~288.0 eV. All of this should result in a theoretical O/C ratio of 0.56 and a change in the ratio between the areas of the C_286.7_/C_288.2_ peaks, which from 5:1 should (considering that environments ii and iii can be resolved with difficulty) shift to 13:1. Curve fitting processing on the C1s peak obtained experimentally using the four chemical environments mentioned above led to the following concentrations: (i) hydrocarbon component and hydrocarbon contamination (285 eV) 23.2%, (ii) chemical environment (286.7 eV) 63.4%, (iii) chemical environment (287 eV) 3.29%, and (iv) chemical environment (288.2 eV) 10.17%. The ratio C_286.7+287_/C_288.3_ was 6.55 To determine the yield of the epoxidation reaction, the following expression was used:

$$\frac{C_{286.7+287}}{C_{288.2}}=\frac{\left(moles\;of\;unreacted\;RC\times 5\right)+(mole\;of\;reacted\;RC\times 13)}{\left(moles\;of\;unreacted\;RC\times 1\right)+(mole\;of\;reacted\;RC\times 1)}$$

Considering that the sum of the unreacted and reacted mol. must close at 100, from this analysis, it appears that the functionalization reaction with BDDE affected approximately 19% of the cellulosic units.

#### 3.2.2. Ligand Bioconjugation

**Figure 7:**
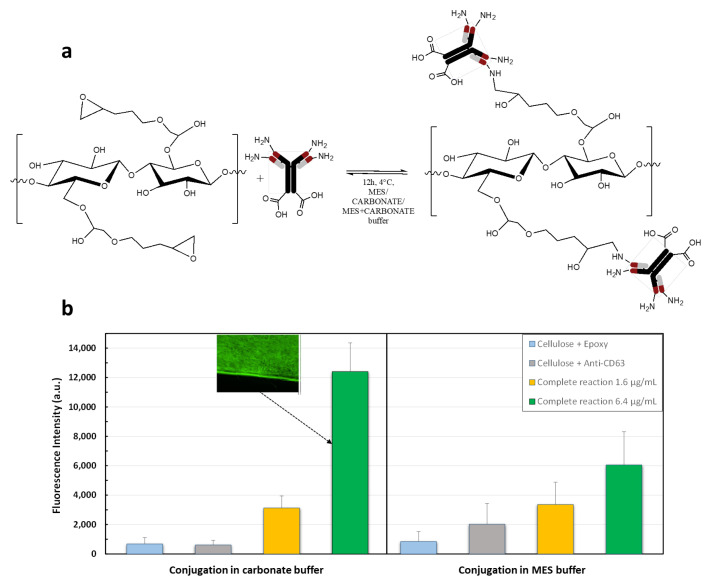
(**a**) Schematic of the bioconjugation of the membranes and (**b**) quantification through fluorescence intensity and a fluorescence image of the fiber with the best conjugation (Ab-FITC concentration of 6.4 µg/mL rinsed in carbonate buffer).

For the immobilization of the FITC labeled anti-CD63 different conjugation conditions were tested to optimize the covalent binding efficiency and to minimize the non-specific interactions between the antibody and the epoxidated RC fibers. In particular, the antibody was tested at concentrations of 1.6 µg/mL and 6.4 µg/m using two different buffers: (I) carbonate buffer at pH 9.5 and (II) MES buffer at pH 5.0. The bioconjugation reaction scheme is shown in Figure 7a.

Negative controls were performed to evaluate the non-specific binding of the Ab-FITC on the surface of the functionalized RC. The results showed an increase in the fluorescence intensity with the Ab-FITC concentration when the samples were rinsed in MES buffer, as reported in Figure 7b on the right panel. As expected, the FITC fluorescence intensity of negative controls was not negligible, but the signal is still lower than the one of the complete reactions performed with 1.6 µg/mL of antibody concentration. The signal at higher antibody concentration (6.4 µg/mL) was higher, but not as expected. However, when the membranes were rinsed in carbonate buffer, as shown in Figure 7b on the left panel, the negative controls were still non-negligible, but below 1000 a.u., while the signal of the complete reactions increased proportionally with the antibody concentration. Indeed the ratio of the fluorescence signal measured at the antibody concentration of 6.4 µg/mL (12,412 a.u.) divided by the fluorescence signal measured at the antibody concentration of 1.6 µg/mL (3132 a.u.) matches the antibody concentration ratio (i.e., 12,412/3132 = 3.96 which is almost the same as 6.4/1.6 = 4).

Therefore, the most favorable condition for the antibody conjugation was rinsing in carbonate buffer at pH 9.5. with the Ab-FITC concentration of 6.4 µg/mL.

Again, the big error affecting the mean fluorescence intensity of the mats that underwent the complete reaction was probably due to the inhomogeneous functionalization of the fibers which is likely to occur when heterogeneous reactions are performed.

### 3.3. Adsorption of EVs on Affinity Membranes

**Figure 8:**
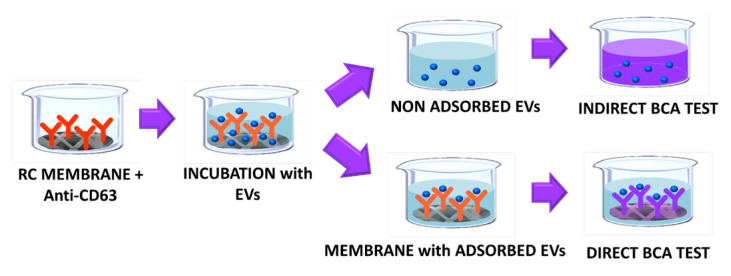
Adsorption test and scheme of the BCA quantification of EVs.

The batch adsorption of EVs on the affinity membranes was quantified using the BCA assay on both the membranes and on the EV solution. The BCA test allows the colorimetric detection and quantification of total proteins and it can be used as a surrogate indirect measure of the EVs concentration. The BCA assay can also be used to determine the total protein adsorbed on a membrane surface as demonstrated by Algeri et al. [44]. In this work, the assay was used for a preliminary quantification of the mass of EVs adsorbed on the RC membranes conjugated with the anti-CD63 antibody as shown in Figure 8.

The BCA test performed on the sample made only of EVs allowed for the quantification of the initial mass of vesicles before the adsorption test with the membranes, while the test carried out on the liquid solution after adsorption on the membrane was needed to calculate the mass of exosomes absorbed on the membrane. From a mass balance on the liquid solution:

$$m_{ads}=c_{i\;}V_{L}-c_{eq}V_{L}$$

where *m_ads_* is the mass of EVs adsorbed on the membrane, *V_L_* is the volume of the EVs solution that was contacted with the affinity membrane, and *c_i_* and *c_eq_* are the concentration of EVs at the beginning of the experiment and at equilibrium, respectively. The values obtained from the indirect quantification of the mass of EVs onto fibers were compared with the ones obtained from the BCA assay directly performed on the membranes and the results are reported in Table 2, together with the binding capacity of the membranes, *q_ads_* calculated as

$$q_{ads}=\frac{m_{ads}}{V_{mem}}$$

where *m_ads_* is the amount of EVs adsorbed on the membrane and *V_mem_* is the membrane volume.

The amount of EVs adsorbed on the membranes prepared with the different conjugation buffers is of the same order of magnitude, however, the membranes conjugated in carbonate buffer gave slightly higher values, which is consistent with the bioconjugation fluorescence results. The results of the two methods were consistent, except for the quantification of the EVs adsorbed on the membranes bioconjugated in MES buffer. In this case, the difference between the direct and indirect quantification of the mass of EVs adsorbed on the membranes was probably due to the instability of the antibody conjugation after binding with the extracellular vesicle. This hypothesis was supported by the data obtained by the measurement of the mean fluorescence intensity response performed on the epoxidized membranes after the bioconjugation reaction in the MES buffer.

It should be noted that these promising results were obtained with preliminary assays and that it is necessary to perform these tests on a larger number of samples to assess their reproducibility. On the other hand, the consistency of the results obtained with the conjugation in carbonate buffer is a proof of concept that this method could be effectively used to prepare affinity membranes for the purification and/or adsorption of extracellular vesicles. In addition, by looking at the values of binding capacity (Table 2), the membranes conjugated in carbonate buffer gave values around 12–13 mg/mL, which are values comparable to those obtained for protein adsorption on electrospun nanofiber membranes [36]. Moreover, the values obtained are more than 20 times higher than those reported by Neumair et al. for the adsorption of EVs in urine, using a system which was conceptually similar to the one reported in this work. They functionalized a monolith by epoxy activation with anti-CD63 nanobodies and purified EVs present in urine [52]. Indeed, they captured EVs from urine samples that were not previously purified, but such a difference in binding capacity cannot be only attributed to the presence of contaminants, but it is more likely due to a less efficient functionalization of the monolith or to the structure of the monolith, compared to our electrospun membrane.

## 4. Conclusions

The research activities carried out in this work led to the successful preparation of an electrospun regenerated cellulose nanofiber mesh that was epoxidized and functionalized with anti-CD63 antibodies to obtain an affinity membrane for the isolation and purification of extracellular vesicles. The XPS analysis of the epoxidized RC nanofiber meshes revealed a higher reaction yield of 19%. The functionalized RC membranes were then bioconjugated with the FITC-labeled anti-CD63 antibody. Of the two different buffers, MES and carbonate, used for the bioconjugation of the antibody, only the carbonate buffer gave consistent results. In this case, the results showed an increase in fluorescence intensity proportional to the antibody concentration in the immobilization reaction. These results assessed the successful grafting of the anti-CD63 onto the surface of the functionalized RC membranes.

Finally, the affinity membranes were tested for adsorption of extracellular vesicles from platelet lysate and the quantification of the mass of EVs adsorbed on the fibers confirmed the presence of the vesicles on the surface of the affinity membranes. The obtained binding capacities around 12 mg/mL are an excellent result, exceeding similar materials reported in the literature. Although further experiments need to be performed in order to optimize and maximize the degree of functionalization and bioconjugation, the electrospun membranes show great potential in capturing EVs. Therefore, the obtained materials represent a proof of concept that affinity membranes capable of capturing and thus purifying extracellular vesicles can be prepared by this method.

## Figures and Tables

**Table 1:** Results of electrospinning optimization.

			Electrospinning Parameters			
Solvent Ratio DCM/EtOH	Cellulose Acetate	CA conc. (*w*/*v*)	Voltage (kV)	Distance (cm)	Rate (mL/h)	Observations *
90/10	CA ds 2.4	10%	10	15	1.0	Fibers, few beads
		5%	15	15	1.5	Few fibers, many beads
	CA ds 3.0	10%	15	20	1.0	Fibers, few beads
		8%	10	15	2.0	Few fibers, many beads
80/20	CA ds 2.4	10%	10	15	1.5	Fibers, few beads
	CA ds 3.0	10%	10	15	2.0	Fibers, NO beads
		8%	15	15	2.0	Fibers, NO beads
		6%	15	15	2.0	Fibers, NO beads
70/30	CA ds 3.0	10%	15	15	2.0	Fibers, NO beads
		8%	15	17	2.0	Fibers, NO beads
		6%	15	15	2.0	Fibers, NO beads

* All experiments were performed at RT.

**Table 2:** Mass of EVs adsorbed on the membrane.

	Indirect Quantification		Direct Quantification	
Sample	*m_ads_* (mg)	*q_ads_* (mg/mL)	*m_ads_* (mg)	*q_ads_* (mg/mL)
Epoxydized membrane bioconjugated in MES buffer	0.0478	9.91	0.0332	6.88
Epoxydized membrane bioconjugated in carbonated buffer	0.0572	11.9	0.0632	13.1

## Data Availability

Data are available upon request.
